# The role of the lymphatic system in musculoskeletal system health and disease: research progress and future directions

**DOI:** 10.1038/s41413-025-00479-0

**Published:** 2026-01-19

**Authors:** Yan Luo, Jie Song, Shengyuan Zheng, Jianfeng Sun, Gaoming Liu, Zirui Xiao, Michael Opoku, Djandan Tadum Arthur Vithran, Jiaxue Zhou, Wenfeng Xiao, Yusheng Li

**Affiliations:** 1 Department of Orthopedics, Xiangya Hospital, Central South University, Changsha, Hunan China; 2 National Clinical Research Center for Geriatric Disorders, Xiangya Hospital, Central South University, Changsha, Hunan China; 3 Department of Clinical Medicine, Xiangya Medicine School, Central South University, Changsha, Hunan China

**Keywords:** Bone, Pathogenesis

## Abstract

The lymphatic system is widely distributed in skeletal muscles, joints, and skeletal tissues and plays a key role in maintaining immune homeostasis, regulating inflammatory responses, and tissue repair. In recent years, an increasing number of studies have shown that morphological and functional changes in lymphatic vessels are closely associated with the onset and progression of a variety of musculoskeletal disorders (MSDs), such as osteoarthritis (OA), fractures, and muscular dystrophy. However, the specific mechanisms of the lymphatic system’s role in these diseases have not been fully elucidated, and their potential clinical value remains to be thoroughly explored. In this review, we review the recent research progress on the structure, function, and pathophysiological role of the lymphatic system in the musculoskeletal system, and we focus on the association between lymphangiogenesis, dysfunction, and MSDs, and systematically summarize the therapeutic strategies targeting the lymphatic system. In addition, we summarize the limitations of current studies and propose key directions for future research, with a view to providing new ideas for basic research and clinical intervention in MSDs.

## Introduction

Lymphatic vessels are important channels for the return of lymphatic fluid into the bloodstream. Vascular endothelial growth factor C (VEGF-C) regulates lymphatic endothelial cells (LECs) proliferation and migration during embryogenesis, resulting in the formation of lymphatic vessels.^1,2^ Lymphatic vessels are found throughout the body and return to the bloodstream in the subclavian vein after collecting metabolic wastes from tissue cells for immune functions.^3^ Skeletal muscles, bones, and joints play an important role in human production and life, and they are governed by consciousness to enable complex and orderly behavioral movements. In healthy individuals, skeletal muscle is the most abundant tissue in the body.^3^ Bones are among the hardest and toughest tissues in the human body. Joints connect parts of the skeleton, hold the bones in place, and allow for flexible movement of the bones. Skeletal muscles, bones, and joints play a key role in human posture maintenance and movement.^4^ However, in recent years musculoskeletal disorders (MSDs) have become prevalent and have had a significant impact on patients’ quality of life and socio-economics.^5^ According to the World Health Organization’s 2003 Technical Report on the Burden of MSDs, 40% of people over the age of 70 years have osteoarthritis (OA), and 80% of these patients have exercise limitations.^6^ The Global Burden of Diseases, Injuries and Risk Factors (GBD) study indicates an overall increase in the global burden of OA from 1990 to 2019, with an estimated 18 948 965 cases of OA worldwide in 2019.^7^

Since the lymphatic system plays an important immune function in disease development and is closely linked to disease progression, a number of studies on diseases related to the musculoskeletal system have explored possible therapeutic options starting from the lymphatic vessels. Currently in MSDs, rheumatoid arthritis (RA) has more established therapeutic options such as anti-tumor necrosis factor (TNF) therapy, B-cell depletion therapy, vascular endothelial growth factor C/vascular endothelial growth factor receptor 3 (VEGF-C/VEGFR-3) therapy, and inducible nitric oxide synthase (iNOS) inhibitors.^8^ In contrast, research on OA has not been elucidated due to technical difficulties in pathogenesis, and although some of the therapeutic strategies have been borrowed from RA findings, most of them are still in the hypothesis or preliminary experimental stage, with large research gaps.^8^ In addition, the pathogenesis of complex lymphatic anomalies (CLA) is still unclear, and there is a lack of effective therapeutic options.^9^ Progress has been made in the targeted treatment of muscle ischemia, and the identification of some key targets has provided the possibility of precise treatment.^8^ The pathogenesis of muscle atrophy is still under further study, and the research prospect is promising.^10,11^

Recent years have seen recent advances in the study of lymphatic vessels in the musculoskeletal system. Recent studies have shown that specific LECs markers lymphatic vascular endothelial hyaluronan receptor 1 (LYVE1) and podoplanin (PDPN)^12^ have enabled imaging of lymphatic vessels, thus revealing the pattern of distribution of lymphatic vessels in bone, joints and muscle: (1) Lymphatic vessels are absent in the modeling remodeling-active zones of the bone; (2) Lymphatic vessels are present in periosteum and surrounding tissues of normal bone and muscle lymphatic vessels; (3) Lymphatic vessels are present around joints.^13^ Gradually, as research techniques advanced, studies proved the existence of lymphatic vessels in bones. In the latest study, researchers determined the presence of lymphatic vessels in human bone using light film imaging of intact bone components, immunostaining, and 3D imaging.^14^ It is a finding that overturns the earlier perception that lymphatic vessels do not exist in bone and provides a new perspective for understanding bone tissue homeostasis and the pathogenesis of bone disease. This groundbreaking study reveals the presence of lymphatic vessels in healthy intervertebral discs (IVDs) through spatial transcriptomics and immunohistochemistry, challenging the long-held dogma that adult IVDs are avascular. The findings parallel recent advancements in musculoskeletal research, such as the discovery of lymphatic vessels in human bone (via light-sheet imaging and 3D analysis), which similarly overturned traditional views on tissue vascularization.^15^ In the study of musculoskeletal system-related diseases, researchers using VEGF-C/VEGFR-3^16^ and the infrared contrast agent indocyanine green (ICG)-near-infrared (NIR) technique^13^ have found that the generation of lymphatic vessels and the drainage function of lymphatic vessels play an important role in slowing down the progression of OA. In addition to this, in the course of studying lymphatic vessels, researchers have focused on specific genes that are highly correlated with the pathogenesis of lymphedema, and based on these genes, targeted therapies have been investigated.^10^

Although the lymphatic system plays a key role in organismal immunity and homeostasis maintenance, its specific mechanisms in MSDs are not clear. There is currently no systematic review that comprehensively summarizes the role of the lymphatic system in MSDs, especially the specific differences in the different tissue environments of bone, joint, and muscle and their potential therapeutic strategies. Based on this, this review focuses on the development of: (1) The distribution characteristics of lymphatic vessels in different musculoskeletal tissues (bone, muscle, and joints); (2) The mechanisms of the lymphatic system’s role in MSDs; (3) The existence of potential therapeutic strategies based on the functional modulation of lymphatic vessels.

## The structure, physiology, and function of the lymphatic vessel network

### The structure of the lymphatic vessel

**Fig. 1:**
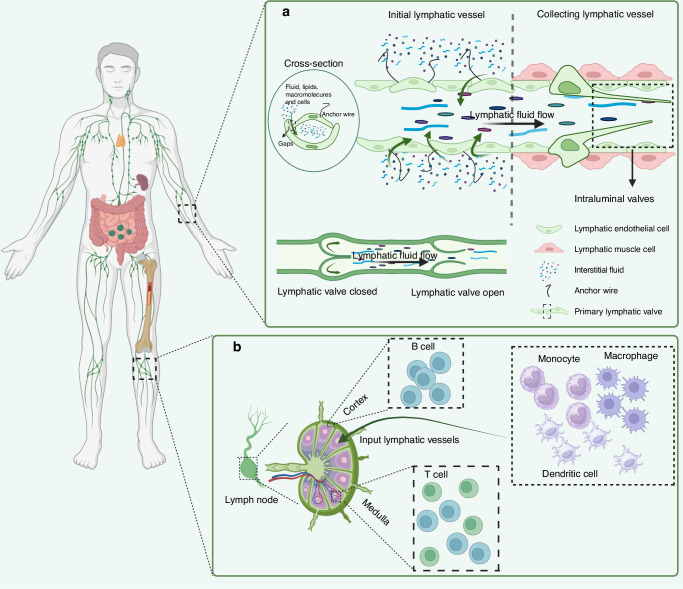
The structure of the lymphatic vessel. **a** The initial lymphatic vessel consists of a single layer of lymphatic endothelial cells (LECs, green) that overlap to form a “primary lymphatic valve” with univalve function, and the intercellular fluid depends on this structure to enter the lumen to form lymphatic fluid. The intraluminal valve prevents backflow of lymphatic fluid. The anchor wires (black filaments) are connected to the LECs, and when they are pressurized by the interstitial fluid (fluid, lipids, macromolecules, and cells), they create gaps between adjacent LECs, whereby fluid enters the lymphatic vessels and participates in the formation of lymphatic fluid. However, this process is still in the speculative stage. Lymphatic muscle cells (pink) are spiral contractile elements regulating fluid propulsion. **b** There are B cells, macrophages, monocytes, dendritic cells, and T cells in the lymphatic nodes. B cells are mainly concentrated in the cortical area of the lymphatic nodes. T cells are mainly located in the paracortical area, and both T and B cells are found in the medullary area. With biofiltration, all monocytes, macrophages and dendritic cells in the lymph fluid remain in the lymphatic nodes

The structure of lymphatic vessels is well recognized **(**Fig. 1**)**. Lymphatic vessels originate from the initial lymphatic vessels [immunostaining characterized as podoplanin-positive/α-smooth muscle actin-negative (PDPN^+^/α-SMA^-^)^8,17–19^], which are also known as terminal lymphatic vessels and lymphatic capillaries. From the initial lymphatic vessel, lymphatic fluid passes sequentially through intermediate lymphatic vessels, afferent lymphatic vessels, lymphatic nodes, efferent lymphatic vessels, and lymphatic trunks (e.g., thoracic duct, right lymphatic vessel, etc.) before finally converging into a vein.^20–23^ Among them, afferent and efferent lymphatic vessels are also collectively known as collecting lymphatic vessels [whose immunostaining characteristics show (PDPN^+^/α-SMA^+^)^8,17–19^].^3^ The morphology of initial lymphatic vessels varies greatly between species and tissues. Initial lymphatic vessels are present in the vicinity of the microcirculation^24^ and consist of a single layer of LECs and a discontinuous basement, with an intraluminal valve (composed mainly of endothelial cells and connective tissue) serving as a demarcation from the next level of lymphatic vessels.^3^ The initial lymphatic vessels are often blind-ended, a structural feature that allows coverage of more tissue surface.^3,8,25^ The cells of the wall overlap to form “primary lymphatic valves” that function as one-way valves and on which the intercellular fluid depends to enter the lumen and form lymphatic fluid.^26^ The wall cells are connected by “button-like” junctions composed of adhesion proteins (VE-calmodulin) and tight junction proteins [Occludin, Claudin-5, Zonula occludens-1 (ZO-1), Endothelial cell-Selective adhesion molecule (ESAM), Junctional adhesion molecule A (JAM-A), etc.].^3,8,24^ In addition, the anchor wires are connected to the surrounding interstitial space to anchor the initial lymphatic vessels, and it is still speculative whether they are related to the formation of lymphatic fluid.^3,27,28^ There are unidirectional valves in the intermediate lymphatic vessels, which act only as conduits for lymph flow and are not involved in lymph formation.^3^ Collecting lymphatic vessels have a continuous layer of basement membranes and lymphatic muscle cells (LMCs). In the collecting lymphatic vessels, there are corresponding “secondary lymphatic valves” called intraluminal valves, each of which consists of a double layer of endothelial cells, which are more numerous and thicker than the “primary lymphatic valves” and which function as a tandem lymphatic vessel.^3,26^ Immunostaining markers used in the study of lymphatic vessel distribution include LECs and LMCs. Among them, LYVE1,^29,30^ PDPN,^31^ prospero homeobox protein 1 (PROX1)^32^ and VEGFR3^33–35^ were used for LECs immunostaining, and α-SMA was used for LMCs immunostaining.^8,14,36^ Factors such as α-SMA, PDPN, PROX1, and VEGFR3 were found to be positively expressed in pooled lymphatic vessels in immunostaining. Collecting lymphatic vessels pass through afferent lymphatic vessels, where specific immune-associated cells converge into the lymphatic fluid in the lymphatic sinus and eventually return to the bloodstream by the subclavian vein. These ducts form a three-dimensional lymphatic network with a branching structure, which is connected to the veins and accompanied by a network of small arteries, distributed throughout the body.^3^

### The physiology of the lymphatic vessel network

There are important physiological functions behind the formation of lymphatic fluid and the flow of lymphatic fluid through the lymphatic vessels. The “button-like” connections of primary LECs and the structure of anchor wires allow the interstitial fluid around the initial lymphatic vessels, which contains macromolecules or small molecules produced by cellular metabolism, to infiltrate into the lymphatic vessels to form lymphatic fluid.^37^ It has been hypothesized that when the pressure of the interstitial fluid increases, the anchor wires are compressed, so that the interstitial fluid enters lymphatic vessels through the gaps created between LECs and neighboring LECs that are connected to the anchor wires. But the mechanism by which anchor wires play a role in this process lacks scientific evidence.^8,24^

Lymphatic fluid flows through all levels of lymphatic vessels after its formation, and the direction of flow and the flow dynamics are two important factors of lymphatic flow. The structure of the lymphatic valves at all levels in the lymphatic vessels allows lymphatic fluid to flow in a fixed direction and plays a key role in preventing lymphatic reflux.^3,8,21,37–39^ LMCs act as endogenous pumps in driving the flow of lymphatic fluid. Tissue contraction and diastole in the heart, skeletal muscle, chest, and intestinal wall exert an exogenous pump.^40–42^ The endogenous and exogenous pumps push the lymphatic fluid in the lymphatic vessels continuously down to the next level of lymphatic vessels to accomplish the transportation of lymphatic fluid.^37^ These physiologic structures allow for the normal flow of lymphatic fluid through the lymphatic vessels.

### The function of the lymphatic vessel network

In normal tissues, the lymphatic system transports some of the nutrients required by cells and removes excreted wastes, thereby participating in the regulation of body homeostasis.^13,37^ Beyond passive transport, there exists a multi-layered active regulatory relationship between lymphatic vessels and immune cells: LECs actively recruit immune cells such as dendritic cells and T cells by expressing chemokines (e.g., CCL21) and adhesion molecules, and increase permeability to accelerate immune cell migration under inflammatory conditions.^43–45^

For the circulatory system, the lymphatic system can eliminate pathogenic microorganisms from the bloodstream through immunosurveillance and immune responses using immunologically active substances in the lymph fluid and cells involved in the immune response.^46–55^ LECs are directly involved in immune regulation: they influence T cell differentiation and tolerance through antigen presentation;^56^, and they secrete cytokines to coordinate the balance between pro-inflammatory and anti-inflammatory responses.^57,58^ This process is mainly realized in the lymphatic nodes,^59^ where all monocytes, macrophages and dendritic cells in the lymph fluid remain in the lymphatic nodes through biofiltration, at which time T cells, B cells and antigen-presenting cells in the lymphatic nodes sample antigens in the lymph fluid that passes through the lymphatic nodes and function as immune surveillance and immune response. At the same time, the lymphatic nodes can exclude proteins from the lymphatic nodes through mechanical filtration and continue with the lymphatic fluid to the next level of lymphatic vessels.^3,60–62^

The lymphatic system uses lymphatic nodes to remove inflammatory factors and deliver immune cells through the functions of lymphatic drainage and reflux, which is important for maintaining the homeostasis of the internal environment, slowing down the progression of the disease, and recovering from the disease.^13,16^ Impairment of the above functions of the lymphatic system is closely related to the disruption of the homeostatic balance of the organism and to the processes of disease in the organism. This also explains to us the reason why lymphatic hyperplasia is often observed in diseased tissues in many cases. In tissues and organs such as bone, where the presence of lymphatic vessels in normal conditions is controversial, it has also been determined that new lymphatic vessels are generated in bone in pathological conditions such as OA.^3^

## The hierarchical structure and function of the lymphatic system in the musculoskeletal system

### The hierarchical structure and function of the lymphatic system in skeletal muscles

**Fig. 2:**
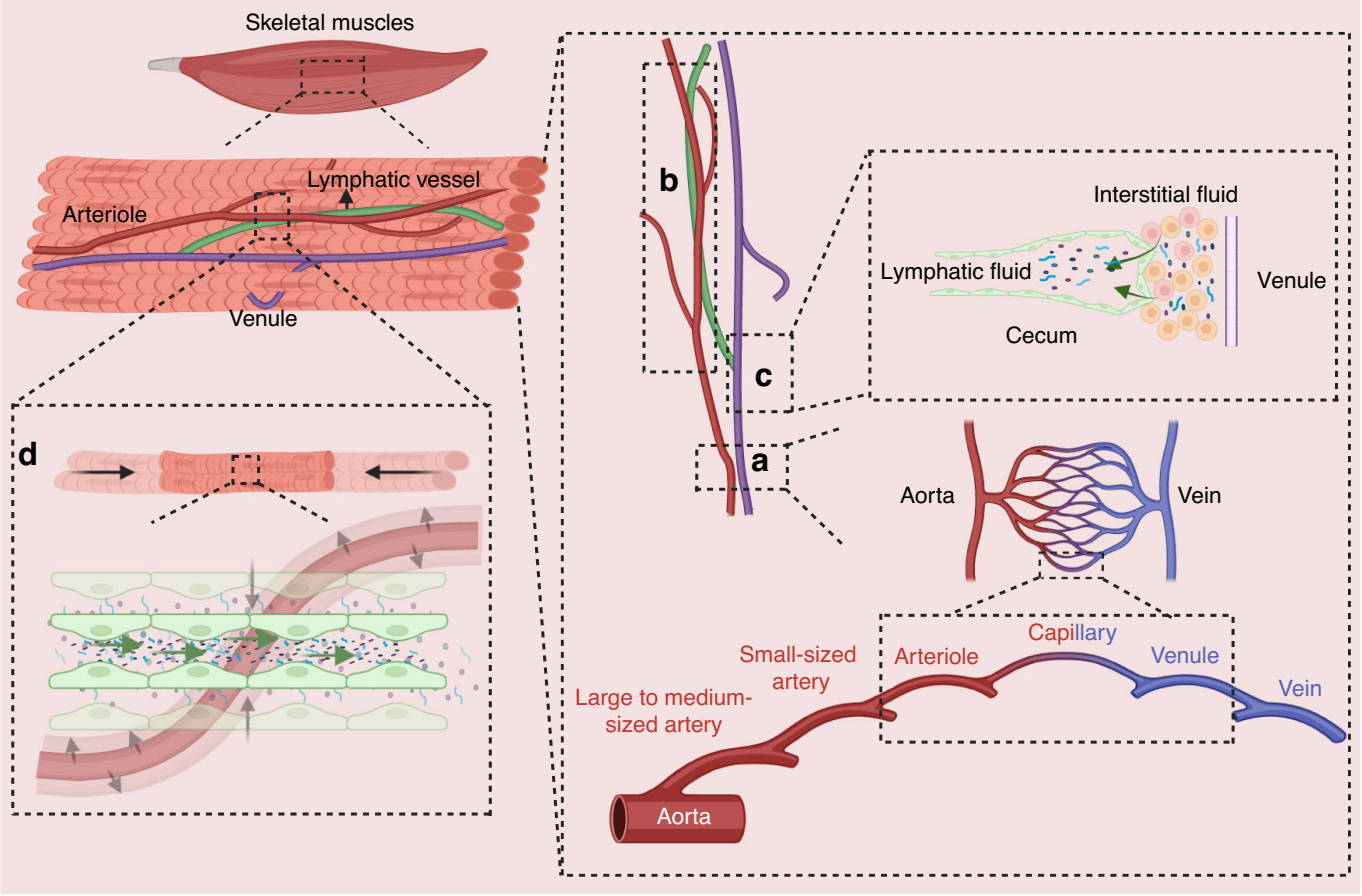
The hierarchical structure and function of lymphatic system in skeletal muscles. **a** Aortas and veins can be connected to each other by a network of capillaries. From the aortas, the veins are connected sequentially by large to medium-sized arteries, small-sized arteries, arterioles, capillaries, and venules. Arterioles and venules in the skeletal muscle shown in the figure are connected by intermediate capillaries. **b** Lymphatic vessels in skeletal muscle are generally accompanied by arterioles. **c** The blind end of a lymphatic vessel (cecum) in skeletal muscle begins at the end of a venule. **d** Arterial blood flow increases during skeletal muscle contraction, and deformation of skeletal muscle and arteries promotes lymphatic fluid flow

With respect to the distribution of lymphatic vessels in skeletal muscle, they initially originate from small veins^63^ in the skeletal muscle and are accompanied by arterioles.^39^ The accompanying arterioles around the initial lymphatics are responsible for providing pulse pressure to supplement the lack of endogenous pumping due to the absence of an initial lymphatic smooth muscle layer, while forming a homogeneous three-dimensional lymphatic network system in skeletal muscle that does not contain lymphatic sinuses **(**Fig. 2**)**. Blood circulation in the body is accelerated after exercise, and there is a significant increase in blood flow in the small arteries that accompany lymphatic vessels in skeletal muscle. Due to the aforementioned properties of lymphatic vessels in skeletal muscle, the flow of lymph in skeletal muscle increases in response to the increase in blood flow in the arteries.^3^^,^^64^ In addition to these properties, the lymphatic vessels in skeletal muscle follow the general distribution of lymphatic vessels, with multiple levels of lymphatic vessels constituting the lymphatic system, with characteristic structures at each level.^13^ In addition to their usual function of transporting substances, lymphatic vessels in skeletal muscle play a role in the development of disease and immunity. When inflammation occurs in bones, joints, or surrounding skeletal muscle, the reflux function of the collecting lymphatic vessels is usually impeded, preventing the lymphatic system from functioning properly in immune surveillance and immune response. Therefore, improving lymphatic return drainage in MSDs can effectively slow down the progression of the diseases.^13^ Skeletal muscle undergoes morphological changes during exercise, and this change affects the lymphatic vessels distributed in it, driving and facilitating the flow of lymphatic fluid.^65^

### The hierarchical structure and function of the lymphatic system in the joint

**Fig. 3:**
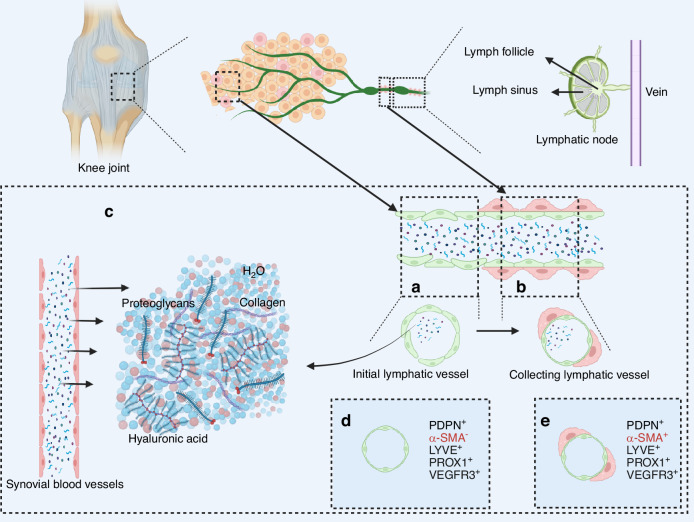
The hierarchical structure and function of the lymphatic system in joint. **a** The initial lymphatic vessels at the joints consist of a single layer of lymphatic endothelial cells (LECs). **b** The collecting lymphatic vessels at the joints consist of LECs in the inner layer and an outermost layer of lymphatic muscle cells (LMCs). **c** Synovial fluid at the joint is formed by exudation of blood from synovial vessels and lymphatic fluid from lymphatic vessels and consists mainly of water, proteoglycans, collagen, and hyaluronic acid. **d** Immunostaining characteristics of initial lymphatic vessels: PDPN positive, α-smooth muscle actin (α-SMA) negative, lymphatic vascular endothelial hyaluronan receptor (LYVE) positive, prospero homeobox protein 1 (PROX1) positive, vascular endothelial growth factor receptor 3 (VEGFR3) positive. **e** Immunostaining characteristics of collecting lymphatic vessels: PDPN positive, α-SMA positive, LYVE positive, PROX1 positive, VEGFR3 positive

The synovium is an important part of the joint that connects the bones at both ends of the joint, and the lymphatic vessels at the joint are also distributed in the synovium and play an important physiological function **(**Fig. 3**)**. The synovium is divided into two layers, containing a variety of macrophages and fibroblasts, and the distribution of lymphatic vessels varies greatly depends on the type and number of cells in the inner and outer layers of the synovium.^66^ Using markers such as 5‘-nucleotidase, cluster of differentiation (CD) 31, CD34, pathologic autopsy leiden endothelium (PAL-E) and multiple immunostaining labeling techniques, researchers have found that the outer layer of synovium, which covers the inner articular cavity, has scattered lymphatics and that there is no presence of lymphatics in the inner layer of the synovium or in the articular cartilage.^16^ LECs at the joints also express specific factors, and lymphatic vessels in the synovium can be recognized by positive expression of LYVE1, PDPN, PROX1, and VEGFR-3.^8^ Lymphatic vessels in the synovium are dependent on pre-existing lymphatic vessels induced through the VEGF-C/VEGFR-3 pathway, which regulates lymphangiogenesis by promoting cell proliferation and inhibiting apoptosis. VEGF-C can be derived from synovial macrophages, so the lymphatic system in the joints is mainly concentrated in the synovial membrane of the joints and is structurally similar to the lymphatic system of the general tissues. The lymphatic system in the synovium consists of lymphatic capillaries and lymphatic vessels. The “button-like” connections of LECs and the endogenous pumping action of LMCs allow the lymphatic vessels in the joints to collect fluids in the joint cavity, drain metabolites and inflammatory factors from the cells in the joints, and then flow with the lymphatic fluid to the lymphatic nodes. The flow of lymphatic fluid removes inflammatory factors in the lymphatic nodes and elsewhere.^16,67,68^ The drainage function of lymphatic vessels influences joint homeostasis. When lymphatic drainage is impaired, it causes infiltration of inflammatory cells in the joints and an increase in the expression of inflammatory factors, leading to inflammation and lymphatic hyperplasia, which in turn leads to the development of diseases such as OA.^17,69^ The specific mechanism of action of this process has not yet been clarified.^70^

### The hierarchical structure and function of the lymphatic system in the bone

Bone and skeletal muscle are closely related, and skeletal muscle is dependent on bone in the human body. Lymphatic vessels in skeletal muscle are more well established than in skeletal muscle, but lymphatic vessels in bone are more difficult to study. In studies on the distribution of lymphatic vessels in bone and skeletal muscle, it is clear that lymphatic vessels proliferate when the musculoskeletal system is in a state of inflammation or disease. For the normal musculoskeletal system, researchers used the lymphatic endothelial cell-specific expression factors LYVE1 and PDPN as evidence for the presence of lymphatic vessels in tests using rats and mice. The results showed that the presence of lymphatic vessels was not detected in normal bone, but the tissues surrounding the bone, such as periosteal fibrous tissue, fat, and skeletal muscle, had a distribution of lymphatic vessels. In some of the existing studies, it is very difficult to detect lymphatic vessels in bones due to the limited technical conditions before,^71–73^ so it is questionable whether there is a distribution of lymphatic vessels in normal bones or it is believed that lymphatic vessels do not exist in normal healthy bones, and that lymphatic vessel proliferation is only induced when OA or other MSDs occur that causes a distribution of lymphatic vessels in the bone.^2^

The existence of lymphatic vessels in bone has been controversial.^74–83^ And in recent years, the latest research results have broken the previous situation and created a new understanding.^14^ Using advanced light-sheet imaging technology, researchers identified LYVE1-positive lymphatic vessels predominantly in cortical bone and to a lesser extent in the bone marrow cavity of long bones, while excluding interference from mesenchymal cells and macrophages. The presence was further confirmed through multiple complementary techniques, including Evans blue staining, immunostaining for PDPN and PROX1, as well as flow cytometry analysis.^14^ However, a contrasting study using Prox1-tdTomato transgenic mice and polyethylene glycol-associated solvent system (PEGASOS) tissue clearing technology demonstrated that lymphatics are absent in both embryonic and adult bones under physiological conditions, though they form a rich network surrounding the bone.^84^ This study provides conclusive evidence overcoming previous contradictory reports about lymphatic vessels in bone, demonstrating their existence in both cortical and trabecular bone regions through the detection of specific LECs markers. These bone-associated lymphatic vessels (BALVs) form an intricate network that is interdigitated with blood vessels throughout the musculoskeletal system.^85^ Further experimental results revealed that genotoxic stress from radiotherapy and chemotherapy stimulates the proliferation of LECs, leading to lymphatic vessel expansion.^14^ This expansion occurs primarily in cortical bone and promotes myosin heavy chain 11 (Myh11) positive pericyte proliferation, thereby enhancing bone regeneration and hematopoiesis while mitigating radiation and chemotherapy toxicity. ^14^ However, it is worth noting that aging-associated dysfunction of collecting lymphatic vessels impairs their physiological roles, including lymphatic drainage, immune surveillance, and inflammatory regulation. This dysfunction can lead to impaired immune cell trafficking, chronic low-grade inflammation, and tissue degeneration—processes implicated in OA pathogenesis.^70^ Similar to synovial lymphangiogenesis, skeletal lymphangiogenesis is primarily regulated by VEGF-C/VEGFR3 signaling. Beyond this pathway, interleukin 6 (IL6) has been identified as another critical mediator of lymphatic vessel formation in bone, as demonstrated through functional experiments.^14^

### The hierarchical structure and function of the lymphatic system in the intervertebral disc

The study of lymphatic vessels in the intervertebral disc has been an area of high potential in recent years. While conventional wisdom held that normal IVDs lack lymphatic vessels—with none detected in the nucleus pulposus or annulus fibrosus (AF), though present in surrounding ligaments^86,87^ —recent breakthroughs have overturned this paradigm.^15^ By demonstrating that lymphatic vessels regulate immune cell recycling and inflammation in IVDs—and that their decline correlates with degeneration—this work not only redefines the pathophysiology of disc degeneration but also positions lymphatic modulation as a novel therapeutic target, echoing its emerging role in bone homeostasis and disease.^15^ These findings align with observations that lymphatic vessels appear in degenerated discs (e.g., herniation) when fibrous tissue invasion occurs upon contact with surrounding soft tissues.^86^ While Khadanovich et al.^88^ anatomically mapped the thoracic duct’s consistent right-sided course and cisterna chyli’s left-sided predominance (70% prevalence) at lumbar levels. These complementary findings redefine IVDs' vascularity across scales. Crucially, the anatomical study revealed that these microscopic changes occur in a specific spatial context: the thoracic duct overlaps with the right diaphragmatic crus at L2-L3 vertebrae, while the cisterna chyli overlaps with the left crus at L3—creating high-risk zones for iatrogenic injury during spinal procedures.^88^ Their presence enables critical lymphatic functions: drainage and immune regulation, which may mitigate disease progression.^89^ Five lymphatic vessel-associated genes (LAGs), mesenchymal-epithelial transition factor (MET), hedgehog-interacting protein (HHIP), sprouty receptor tyrosine kinase signaling antagonist 1 (SPRY1), colony-stimulating factor 1 (CSF1), and thymocyte selection-associated high mobility group box (TOX), were utilized in the study to identify and diagnose patients with intervertebral disc degeneration (IVDD). There are 50 potential drugs targeting MET and 2 potential drugs targeting CSF1, which offer the possibility of targeted therapy.^90^

## Roles of lymphatic vessels in musculoskeletal system diseases

### Roles of lymphatic vessels in RA

**Fig. 4:**
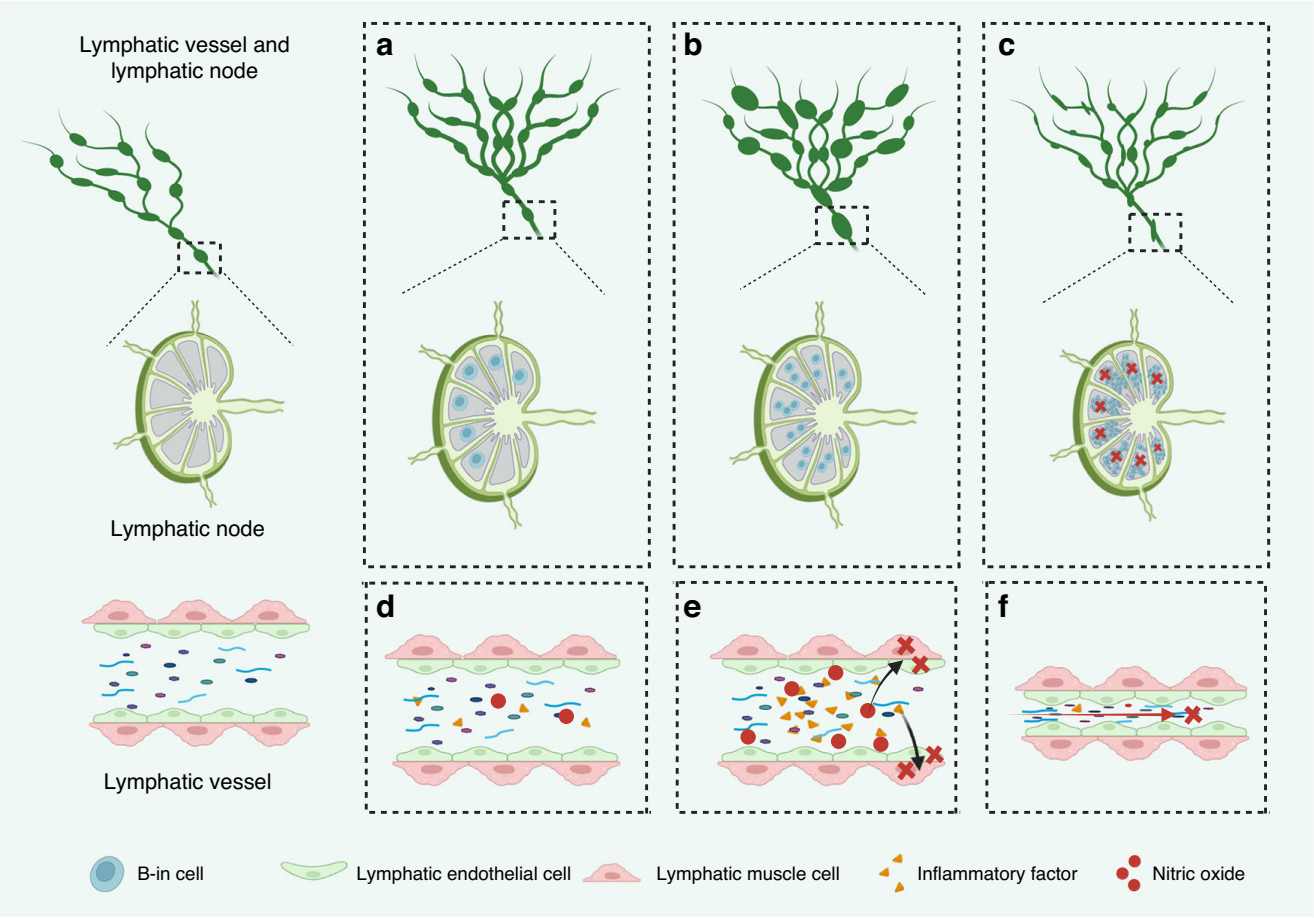
Roles of lymphatic vessels in rheumatoid arthritis (RA). **a** Dilated and hyperplastic lymphatic vessels with B-in cells in the lymphatic nodes in the initial stage of RA. **b** With the aggravation of RA, the lymphatic nodes first show a tendency to increase in size. **c** With further aggravation, the lymphatic nodes become smaller and enter the collapsed stage, where B-in cells obstruct the lymphatic nodes. **d** In the initial stage of RA, inflammatory factors and macrophage inducible nitric oxide synthase (iNOS) induced production of nitric oxide (NO) appear in the lymphatic vessels. **e** Inflammatory factors and NO flowing in the lymphatic vessels damage lymphatic endothelial cells (LECs) and lymphatic muscle cells (LMCs). **f** Damage to LECs and LMCs impairs lymphatic drainage

The structural and functional alterations in synovial lymphatic vessels are fundamentally interconnected with the pathological progression of RA, a systemic autoimmune disorder characterized by chronic synovitis and progressive joint destruction.^8,91^ Recent studies using lymph nodes (LNs) biopsies reveal that neutrophils, typically absent in healthy LNs, significantly accumulate in RA patients’ lymph nodes through lymphatic transport, suggesting active neutrophil trafficking via lymphatic vessels during inflammation.^92^ During the initial stages of RA, before overt lymphatic contraction dysfunction becomes apparent, lymphangiogenesis is upregulated via the VEGF-C/VEGFR3 signaling pathway, primarily driven by pro-inflammatory cytokines (e.g., TNF-α, IL-1β, and IL-6) secreted by synovial fibroblasts and infiltrating macrophages.^93–95^ Concurrently, a distinct population of CD23^+^/CD21^hi^ B cells (B-in cells) accumulates in draining lymph nodes (DLNs).^96–98^ These adaptive changes facilitate the clearance of metabolic byproducts and inflammatory mediators from the joints, temporarily mitigating inflammatory progression (Fig. 4).

As RA progresses to chronic stages, the sustained inflammatory microenvironment exerts dual damaging effects on lymphatic vasculature: (1) inflammatory mediators within lymph fluid directly impair both LECs and LMCs,^68,69,99^, and (2) macrophage-derived iNOS induction leads to excessive NO production, which disrupts normal lymphatic contractility through cGMP-dependent mechanisms.^93,100^ The discovery of neutrophil-specific protease recombinant cathepsin G (CTSG) overexpression in LN biopsies further supports that lymphatic vessels serve as conduits for inflammatory cell trafficking, potentially exacerbating LN dysfunction.^92^ This lymphatic dysfunction creates a vicious cycle where impaired drainage capacity results in progressive accumulation of inflammatory factors, further exacerbating synovitis and tissue damage. The VEGF-C/VEGFR3 signaling pathway continues to influence disease progression, as demonstrated in TNF-Tg mouse models, where lymphatic expansion phase features increased lymphatic density through VEGF-C/VEGFR3 upregulation, temporarily ameliorating inflammation through combined effects with B-in cell accumulation in DLNs.^93,101^

The subsequent lymphatic contractile phase marks a critical transition in RA pathogenesis, characterized by structural damage to collecting lymphatic vessels from persistent inflammatory insults, manifested by vessel wall abnormalities and luminal narrowing.^69,99^ This leads to the characteristic biphasic LNs dynamics - initial enlargement followed by progressive collapse - resulting from mechanical obstruction by accumulated B-in cells and impaired lymphatic flow.^96,97^ The eventual collapse phase represents end-stage lymphatic failure, featuring complete drainage dysfunction, lymph stasis, and uncontrolled inflammation amplification, which correlates with radiographic joint destruction and clinical disease progression.^8,68^ Notably, CD11^b+^ macrophages play dual roles in this process by both modulating lymphangiogenesis and differentiating into osteoclast precursors, thereby directly contributing to periarticular bone erosion.^98,102,103^

### Roles of lymphatic vessels in OA

**Fig. 5:**
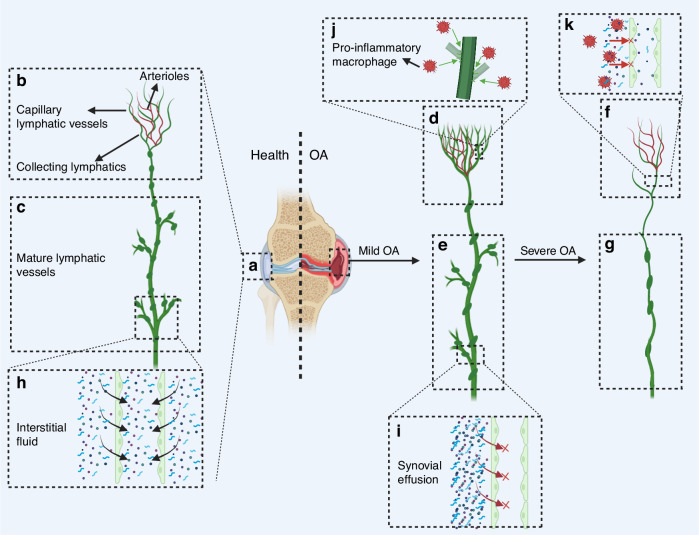
Roles of lymphatic vessels in osteoarthritis (OA). **a** Lymphatic vessels in normal joints include **b** capillary lymphatic vessels with arterioles accompanying, and **c** mature lymphatic vessels that perform drainage functions. **d** The number of capillary lymphatic vessels is increased in mild OA. **e** Decreased number of mature lymphatic vessels in mild OA. **f** Decreased number of capillary lymphatic vessels in severe OA. **g** A significant decrease in the number of mature lymphatic vessels in severe OA. **h** It is the mature lymphatic vessels that have the function of drainage. **i** Fluid accumulation in the joint at the onset of OA interferes with the lymphatic drainage function. **j** Pro-inflammatory macrophages in the diseased site of OA stimulate the proliferation of collecting lymphatic vessels. **k** Pro-inflammatory macrophages in the diseased site of OA reduce the drainage of the collecting lymphatic vessels

The development and progression of OA is intimately associated with dynamic alterations in synovial lymphatic structure and function, representing a critical but understudied aspect of joint homeostasis dysregulation (Fig. 5**)**.^16,68^ Unlike the primarily immune-driven pathology of RA, OA involves a complex interplay between mechanical stress, low-grade inflammation, and metabolic factors that collectively influence lymphatic behavior (Table 1).^8,104,105^ In the early stages of OA, inflammatory factors (TNF-α, IL-1β) drive lymphatic vessel proliferation through the VEGF-C/VEGFR-3 signaling pathway, enhancing the clearance capacity of inflammatory mediators.^8,106,107^ Lymphatic vessels augment their drainage function through compensatory dilation, alleviating intra-articular pressure,^106,108^ during which time the draining LNs are often enlarged.^99,100^ Recent evidence suggests that lymphatic vessels and draining LNs function as an integrated unit in OA pathogenesis. While lymphatic vessels mediate initial inflammatory cell transport, subsequent T-cell retention in LNs—as demonstrated in load-induced OA models—may exacerbate joint damage through two mechanisms: (1) impaired immune surveillance due to reduced T-cell egress, and (2) sustained pro-inflammatory cytokine production (e.g., TNF-α/IL-6) that circulates back to the joint via lymphatic-venous anastomoses. This explains why sphingosine-1-phosphate (S1P) receptor inhibitors (which restore T-cell migration) attenuate cartilage degeneration despite not directly targeting lymphatic vessels.^109^

**Table 1:** Comparative analysis of lymphatic system involvement in RA vs. OA

Comparison dimension	RA	OA	Shared challenges & future directions	References
Disease nature	Autoimmune disorder with systemic chronic inflammation	Degenerative joint disease with localized low-grade inflammation	Need to develop disease-specific lymphatic evaluation criteria	^105,157,158^
Inflammatory status	High-grade inflammation with massive immune cell infiltration	Mild inflammation primarily driven by mechanical stress responses	Establishing quantitative relationships between inflammation levels and lymphatic dysfunction	^91,157,159,160^
Lymphangiogenesis features	Early-stage dramatic increase via strong VEGF-C/VEGFR3 signaling activation	Compensatory early increase but late decline with age-dependent VEGF-C reduction	Optimizing lymphangiogenesis modulation strategies to balance growth and functional maintenance	^93,101,107,161^
Lymph node dynamics	Biphasic changes: Expansion phase (volume increase) → Collapse phase (volume decrease)	Possible early enlargement with minimal late-stage changes	Developing dynamic lymph node imaging technologies	^96,97,104,162^
Key dysfunction mechanisms	iNOS/NO pathway overactivation impairing LMC contraction + B-in cell sinus obstruction	Oxidative stress and weakened VEGFR3 signaling damaging LECs + mechanical stress effects	Exploring combined iNOS inhibitors and antioxidant therapies	^69,99,107^
Immune cell Interactions	CD11b+ macrophages promoting lymphangiogenesis/osteoclastogenesis + B-in cell aggregation	M1/M2 macrophage imbalance with M1-dominant suppression of lymphatic function	Single-cell mapping of immune cell-lymphatic interactions	^66,98,102,163^
Molecular regulation	Dominant VEGF-C/VEGFR3 and NF-κB/iNOS signaling pathways	Attenuated VEGF-C/VEGFR3 signaling + enhanced oxidative/mechanical stress signals	Multi-omics integration of regulatory networks	^93,107^
Spatiotemporal distribution	Increased synovial capillaries/collecting vessels but late-stage collecting vessel dysfunction	Early capillary increase → Progressive mature vessel loss → Collecting vessel decline	Advanced spatiotemporal tracking technologies	^17,18,104^
Bone/Joint damage link	Direct promotion of bone erosion via osteoclast activation	Indirect metabolic effects through inflammatory mediator retention	Elucidating lymphatic-bone metabolic axis mechanisms	^98,115,164^
Current therapeutic targets	Anti-TNF therapy, B-cell depletion, VEGFR3 agonists, iNOS inhibitors	VEGF-C supplementation, macrophage polarization modulation, mechanical load adjustment	Targeted drug delivery systems for enhanced specificity	^66,107,165,166^
Technical limitations	Difficulties in real-time monitoring of lymphatic contractile function	Challenges distinguishing lymphatics from blood vessels	High-resolution lymphatic imaging development	^17,167^
Translational challenges	Balancing lymphangiogenesis promotion vs. inflammation control	Overcoming age-related lymphatic regeneration barriers	Humanized animal models and organoid platforms	^101,161^
Personalized therapy	Stage-adapted approaches based on lymphatic status	Etiology-specific strategies (post-traumatic/metabolic/age-related subtypes)	Biomarker systems for lymphatic function assessment	^99,107,165^
Cutting-edge research	Decoding lymphatic-immune-bone erosion regulatory networks	Mechanical stress-lymph function-cartilage degeneration axis studies	Gene editing and cell therapy for lymphatic reconstruction	^14,168,169^

*RA* rheumatoid arthritis, *OA* osteoarthritis, *VEGF-C/VEGFR3* vascular endothelial growth factor C/vascular endothelial growth factor receptor 3, *VEGF-C* vascular endothelial growth factor C, *iNOS* inducible nitric oxide synthase, *LECs* lymphatic endothelial cells, *LMCs* lymphatic muscle cells, *CD* cluster of differentiation, *NF-κB* nuclear factor-κB, *TNF* anti-tumor necrosis factor

Current research faces significant challenges in elucidating OA lymphatic pathophysiology, primarily due to technical limitations in distinguishing lymphatic subtypes within synovial tissues where they coexist with blood vessels and are obscured by effusion and hyperplasia.^17,18^ Advanced whole-slide imaging systems have revealed spatial heterogeneity, demonstrating that mild OA correlates with increased capillary lymphatics, while progressive disease leads to a marked reduction in mature collecting vessels.^17,18^ As OA progresses, accumulated inflammatory factors (e.g., TNF-α) and collagen fragments disrupt LECs function: (1) TNF-α inhibits lymphatic contraction via nuclear factor-κB (NF-κB)-iNOS signaling^93,110^; (2) Oxidative stress and impaired VEGFR-3 signaling induce lymphatic atrophy^107,111,112^, and (3) age-related decline in VEGF-C disrupts VEGFR3 maintenance signaling.^107^ Postmortem analyses confirm decreased synovial lymphatic density in advanced OA, though this may reflect impaired lymphangiogenesis rather than absolute vessel loss, given the competing effects of synovial expansion.^12,17,104^

The functional consequences of lymphatic impairment create a self-perpetuating cycle of joint degeneration. Mature lymphatics normally mediate macromolecular drainage, but their dysfunction leads to the accumulation of cartilage-degrading enzymes (matrix metalloproteinases (MMPs), a disintegrin and metalloproteinase with thrombospondin motifs (ADAMTS)) and pro-inflammatory cytokines.^106,113–116^ While the molecular mechanisms driving the reduction in mature lymphatic vessels remain incompletely understood, current evidence suggests involvement of inflammatory cytokine-mediated endothelial dysfunction and impaired VEGF-C/VEGFR3 signaling.^17,117,118^ Lymphatic dysfunction potentially affects bone metabolism through indirect pathways: retained pro-inflammatory cytokines (e.g., TNF-α) can promote osteoclastogenesis via receptor activator of nuclear factor kappa-B ligand (RANKL) signaling, while IL-6 exhibits dual roles—both promoting osteoclast differentiation and suppressing osteoblast activity in a context-dependent manner.^119,120^ For the collecting lymphatic vessels, on the one hand, pro-inflammatory macrophages at the site of inflammation reduce the drainage effect of the surrounding collecting lymphatic vessels. On the other hand, anti-inflammatory macrophages stimulate the proliferation of the collecting lymphatic vessels, which leads to an increase in the number of lymphatics. However, ultimately, the draining effect of lymphatic vessels was still significantly disrupted. Some specific influencing factors, such as TNF-α, IL-1, and IL-6, made it possible to study the specific therapeutic mechanism.^8^

### Roles of lymphatic vessels in other bone diseases

The potential roles of lymphatic vessels in bone diseases remain an emerging area of research. While current evidence primarily comes from acute bone injury models, fractures, and tooth extractions, with a notable lack of studies on chronic bone diseases like osteoporosis, these findings provide preliminary insights into lymphatic-bone crosstalk. Recent studies demonstrate that Vegfc-expressing mesenchymal progenitor cells (MPCs) not only stimulate lymphangiogenesis at musculoskeletal injury sites but also directly differentiate into heterotopic bone, revealing a dual role in both lymphatic network expansion and pathological ossification.^121^

The skeletal lymphatic system serves as a dynamic regulator of bone homeostasis, with its strategic positioning along fracture calluses and marrow sinuses being particularly critical for bone repair.^85^ Interestingly, while lymphatic hypoplasia in Vegfr3wt/Chy mice does not impair fracture healing, complete lymphatic absence in Vegfr3Chy/Chy embryos similarly shows no developmental bone defects, indicating compensatory mechanisms may exist when lymphatics are deficient.^84^ In contrast to physiological conditions, traumatic musculoskeletal injury triggers robust lymphangiogenesis that parallels but does not invade heterotopic ossification (HO) lesions, suggesting spatial regulation of lymphatic invasion.^121^ In fracture healing models, lymphangiogenesis around bone implants coincides with improved bone regeneration.^122^ However, lymphatic dysfunction can exacerbate pathological changes: lymph platelet thrombi (LPT) frequently obstruct the drainage of subcapsular sinuses and collecting ducts after fractures.^85^ Conversely, impaired lymphatic drainage exacerbates fracture healing delays, as demonstrated by VEGFR3 inhibition (SAR131675), which reduces lymphatic clearance and popliteal lymph node (PLN) volume, leading to edema, pain, and poor callus formation. Anti-podoplanin (PDPN) neutralizing antibodies mitigate LPT, enhance drainage, and improve bone repair by unblocking lymphatic vessels and reducing inflammation.^123^ The dual capacity of this system in regenerative expansion and pathological obstruction underscores its significant role in bone physiology and pathology.

Beyond injury repair, lymphatic vessels also regulate inflammatory bone loss. In periprosthetic osteolysis, lymphatic activation via VEGF-C/VEGFR3 signaling counteracts osteoclast differentiation and bone resorption induced by titanium particles or inflammatory cytokines (e.g., TNF-α, LPS).^124^ However, aging impairs this protective mechanism due to senescence-associated secretory phenotype (SASP) from adipogenically differentiated mesenchymal stem cells (MSCs), which suppress LECs responsiveness to VEGF-C. JAK inhibitors (e.g., ruxolitinib) can restore lymphatic vessels function in aged mice, offering a therapeutic strategy for osteolysis prevention.^124^

Moreover, in bisphosphonate (BP)-related osteonecrosis of the jaw (BRONJ) models,^125^ impaired lymphatic drainage due to LECs apoptosis correlates with disease exacerbation. Similarly, in tooth extraction models, parathyroid hormone (PTH) stimulates lymphangiogenesis, aligning with its known anabolic effects on bone.^126^ While acute injury models (fracture/tooth extraction) differ from chronic osteoporosis, they collectively imply that lymphatic modulation could be a therapeutic target. We emphasize that current evidence supports lymphatic involvement in bone homeostasis generally, while osteoporosis-specific mechanisms remain hypothetical. This gap highlights an important research frontier where our review aims to stimulate investigation. Future studies should employ established osteoporosis models to clarify whether lymphatic dysfunction contributes to osteoporosis pathogenesis.

### Roles of lymphatic vessels in CLA

The development of CLA is associated with abnormalities of the lymphatic vessels, and each of the disorders in CLA has its own characteristics. Gorham-Stout Disease (GSD), General Lymphatic Anomaly (GLA), Kaposiform Lymphangiomatosis (KLA), and Central Conducting Lymphatic Anomaly (CCLA) are collectively referred to as CLA, which is a serious physical and mental health risk for patients, with a low overall number of patients for whom there is no effective cure. Recent genomic analysis of GSD lesions identified a somatic Kirsten Ratsarcoma Viral Oncogene Homolog (KRAS) c.182 A > G (p.Q61R) mutation at 1% allele frequency in affected bone tissue, representing the first reported pathogenic variant in GSD that activates RAS/ mitogen-activated protein kinase (MAPK) signaling - a known oncogenic driver in vascular anomalies.^127^ Recent studies have identified somatic activating mutations in PIK3CA (including Glu542Lys, Gln546Lys, His1047Arg, and His1047Leu variants) as causative factors in GLA, with these mutations leading to hyperactivation of the PI3K-AKT-mTOR pathway and subsequent lymphatic hyperplasia.^128^ Histopathological correlation showed this KRAS-mutant GSD case exhibited characteristic LECs proliferation, osteoclast activation, and M1/M2 macrophage infiltration, suggesting mutant cells may drive disease through both direct lymphatic hyperplasia and paracrine cytokine signaling.^127^ Case studies have been conducted at the genetic level. For a long time, lymphatic vessels were thought to be absent from the bone, but in recent studies, they have been confirmed,^14^ and during the development of GSD, GLA, and KLA, the normal bone of the patients is resorbed by the invading lymphatic vessels.^129–133^ The proliferation of lymphatic vessels in GSD and KLA is associated with hyperactivation of Rat sarcomavirus and Mitogen-activated kinase (RASMEK) pathway in VEGF-C/VEGFR3 signaling induced by somatic mutations. Recent research demonstrates that hyperactive KRAS/MAPK signaling disrupts lymphatic vessel architecture and function, leading to lymphatic hyperplasia and impaired valve formation, while MEK1/2 inhibition (e.g., trametinib) partially reverses these defects by suppressing extracellular regulated protein kinases (ERK)1/2 phosphorylation and restoring lymphatic maturation gene expression.^134^ Additionally, lineage-tracing studies reveal that bone lymphatic endothelial cells (bLECs) originate from pre-existing Prox1-positive LECs, with osteoclasts playing a critical role in facilitating lymphatic invasion into bone through cortical resorption.^135^ Preclinical evidence shows that rapamycin effectively prevents lymphatic hyperplasia and dysfunction in mouse models expressing active PIK3CA mutations, while clinical observations demonstrate its ability to reduce pain in GLA patients. ^128^ Rapamycin, an mTOR inhibitor, effectively suppresses this pathological lymphangiogenesis in both GSD and GLA mouse models.^135^ New preclinical studies demonstrate that the MEK1/2 inhibitor trametinib selectively blocks VEGF-C-induced ERK1/2 activation in LECs, preventing lymphatic invasion of bone and cortical bone loss in animal models, though its efficacy is limited to early disease stages and shows minimal reversal of established bone lesions—suggesting a potential therapeutic window for early intervention in GSD patients with KRAS mutations.^136^ CCLA is often characterized by lymphatic malformations causing lymphedema.^137,138^ Mutations in the corresponding somatic cells in each disease do not directly lead to bone loss. They cause lymphangiectasia through overactivation of the cell signaling pathway, such as phosphatidylinositol-4,5-bisphosphate 3-kinase catalytic subunit alpha (PIK3CA) pathway and RAS-MEK, and the lymphangiectasia contacting the skeleton progressively lyses the bone and leads to bone loss. However, the process of lymphatic vessel proliferation and lymphatic vessel osteolysis has not yet been elucidated in the molecular mechanism of the specific influencing factors and their mechanism of action.^9^

### Roles of lymphatic vessels in muscular diseases

**Fig. 6:**
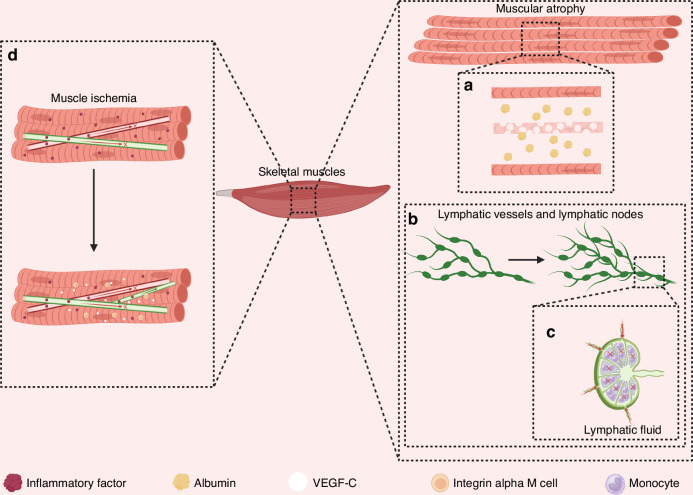
Roles of lymphatic vessels in muscular diseases. **a** In muscle atrophy, the morphology and number of myofibers in skeletal muscle change, with myofibers becoming thin and fewer in number. Myofibers release albumin when damaged. **b** Lymphatic vessels in skeletal muscle appear as new lymphatic buds during muscle atrophy, and these lymphatic buds further generate new lymphatic vessels. **c** Mononuclear cells adhere to lymphatic nodes in muscle atrophy, affecting the drainage function of the lymphatic nodes. **d** Impaired circulation of blood and lymphatic fluids leads to muscle ischemia, and the accumulation of inflammatory factors in the tissues causes inflammation in ischemic muscles. Vascular endothelial growth factor C (VEGF-C) factor produced by integrin alpha M (CD11b) cells can ameliorate muscle ischemia by inducing neolymphangiogenesis

Lymphatic vessels are critical in muscular diseases, particularly in regulating inflammation and tissue homeostasis (Fig. 6). In muscle ischemia, impaired venous return and lymphatic drainage disrupt blood circulation and inflammatory factor clearance, exacerbating inflammation. VEGF-C, produced by integrin alpha M (CD11b) cells, activates the VEGFR-3 pathway to induce lymphangiogenesis, and intramuscular VEGF-C supplementation enhances lymphatic remodeling, mitigating ischemic damage.^10,139^ Muscle atrophy, resulting from malnutrition, disuse, or genetic abnormalities, is associated with lymphangiogenesis, where pre-existing lymphatic vessels develop buds and proliferate via the VEGF-C/VEGFR-3 axis, though the precise mechanisms remain unclear.^10,11^ In muscular dystrophy, myocyte damage leads to albumin release, while monocyte adhesion to peripheral lymphatic nodes with altered extracellular matrix ligand receptor expression contributes to lymphatic dysfunction, LNs atrophy, and impaired drainage, promoting inflammation.^10^

## Targeting the lymphatic vessels as potential treatment strategy in musculoskeletal system

The “button” and anchor wire structures in the initial lymphatic vessels, as well as the intraluminal valve structures and LMCs at all levels of lymphatic vessels, provide the structural basis for the drainage function of the lymphatic system. Only through normal drainage can the lymphatic vessels realize the functions of material transportation, immune surveillance, and immune reaction, and maintain the homeostasis of the organism. Various diseases of the musculoskeletal system show obvious impaired lymphatic drainage in the pathogenesis, accompanied by inflammation. Unclogging lymphatic vessels and increasing the number of lymphatic vessels are both beneficial in restoring their drainage function to slow down the progression of inflammation and MSDs. Currently, different therapeutic programs have been formulated for different MSDs from different perspectives to achieve the goals of increasing the number of lymphatic vessels and preventing lymphatic vessel blockage to enhance the drainage function from different levels and stages of disease development (Table 2).

**Table 2:** Potential therapy by targeting the function of lymphatic vessels in the musculoskeletal system

Musculoskeletal system	Therapy (Drug therapy, Physical therapy, Surgical therapy, Psychotherapy, Genetic therapy)	Drug		Disease model	Therapeutic effect	Reference
Joint	Genetic therapy	Adenovirus vector-mediated VEGFD-ΔNΔC		Patients with refractory angina	Induced cardiac lymphatic growth by lymphangiogenesis and improving their functional maturation.	^170^
	Drug therapy	Adrenomedullin		Patients with refractory angina	Induced cardiac lymphatic growth by lymphangiogenesis and improving their functional maturation.	^170^
	Drug therapy	Anti-TNF treatment	Anti-TNF IgG1 antibody	TNF-Tg mice	Decreased synovial and lymph node volumes without a reduction of lymphatic vessels; Restored lymphatic contractions; Potential enhancement of inflammatory cell egress; Not improved LMCs defects.	^8^
	Drug therapy		Certolizumab pegol	RA patients with active flare of a single wrist or knee	Linear inverse correlation between lymph node volume and joint pain.	^8^
	Drug therapy		Ginsenoside Rg1	TNF-Tg mice	Improved lymphatic drainage; Increased LMCs coverage; Reduced inflammation in LECs and bone erosion.	^8^
	Drug therapy		Etanercept	A rat model of collagen- induced arthritis	More favorable pharmacodynamics than subcutaneous or intravenous administration; Increased lymphatic pumping and reduced swelling of joints.	^8^
	Drug therapy		Etanercept	Inflammatory hand OA patients	Not relieve pain; Radiographic remodeling of subchondral bone.	^8^
	Drug therapy		Adalimumab	Inflammatory hand OA patients	Not show any effect on pain, synovitis or bone marrow lesions.	^8^
	Drug therapy	Proteasome inhibitors	Bortezomib	MLI-induced OA	Decreased cartilage loss; Reduced the expression of inflammatory genes by LECs; Improved lymphatic drainage.	^8^
	Drug therapy	B-cell depletion therapy	Anti CD20 mAbs (18B12 IgG2a)	TNF-Tg mice	Decreased synovial volumes; Converted collapsed DLNs to expanding DLNs; Increased lymphatic clearance Without recovery of the lymphatic pulse Decreased cartilage loss.	^8^
	Drug therapy		Rituximab	RA patients on methotrexate with resistance to TNF inhibitors	Inhibit the progression of structural joint damage; Mild to moderate side effects.	^8^
	Drug therapy		Anti-CD20 antibody (Cy5-αCD20) Rituximab	Collagen-induced arthritis mice Sprague-Dawley rat	IA with a greater B cell depletion in DLNs of joints.	^8^
	Drug therapy	VEGF-C/VEGFR3 treatment	AAV-VEGF-C	TNF-Tg mice	Increased lymphangiogenesis; Improved lymphatic drainage; Attenuated joint tissue damage.	^8^
	Drug therapy		VEGF-C156S	Age-Related OA	Improved lymphatic drainage; Attenuated joint tissue damage.	^8^
	Drug therapy	iNOS inhibitors	Ferulic acid L-NIL L-NAME	TNF-Tg mice	Improved lymphatic drainage; Restored lymphatic contractions; Attenuated joint tissue damage.	^8^
	Drug therapy		Fang-Ji-Huang-Qi-Tang decoction	Collagen- induced arthritis mice	Increased lymphangiogenesis; Improved lymphatic drainage; Attenuated joint tissue damage.	^8^
	Drug therapy		Du-Huo-Ji- Sheng-Tang decoction	TNF-Tg mice Zebrafish	Increased lymphangiogenesis Improved lymphatic drainage; Attenuated joint tissue damage.	^8^
	Drug therapy		Total saponins of Panax notoginseng	TNF-Tg mice	Prevented LMCs apoptosis; Improved lymphatic drainage; Attenuated joint tissue damage.	^8^
	Drug therapy		Cindunistat (SD-6010)	Symptomatic knee OA patients (KLG 2 or 3)	Less joint space narrowing KLG2 patients during early stage of treatment; Not slow OA progression in KLG3 patients.	^8^
	Drug therapy		GW274150	RA patients with DAS28 scores ≥4.0	A trend towards reduction in synovial thickness and vascularity without statistically significant.	^8^
Bone	Drug therapy	FGF9		HO mice	Inhibited heterotopic bone formation	^142^
	Drug therapy	Trametinib		GSD mice	Inhibited of lymphatic valve regression and chylothorax	^141^
Muscle	Drug therapy	VEGF-C		Rabbit hindlimb skeletal muscle	Enhanced the lymphatic drainage and venous reflux function, eliminate the muscle tissue inflammation, and improve the blood supply conditions	^10^

*VEGFD* vascular endothelial growth factor D, *TNF* tumor necrosis factor, *LMC* lymphatic muscle cell, *OA* osteoarthritis, *RA* rheumatoid arthritis, *MLI* meniscal ligamentous injury, *AAV* adeno-associated virus, *VEGF-C* vascular endothelial growth factor C, *IA* intra-articular, *KLG* kellgren and lawrence grade, *HO* heterotopic ossification, *GSD* Gorham-Stout disease, *FGF9* fibroblast growth factor 9, *iNOS* inducible nitric oxide synthase, *L-NAME* Nω-nitro-L-arginine methyl ester, *L-NIL* L-N6-(1-iminoethyl) lysine 5-tetrazole-amide

### Targeting the lymphatic vessels as a potential treatment strategy in joint

There are similarities in the clinical treatment of RA and OA, but at the same time, there are also different treatment directions for each. There are many approaches to the treatment of RA, and some drugs have shown good therapeutic effects in clinical use, while others are still in the stage of animal testing or have yet to be experimentally verified after theoretical proof.^8^ Recent studies demonstrate that in situ size amplification of nanoparticles (e.g., PD5NPs) through myeloperoxidase (MPO)-responsive aggregation can effectively reduce lymphatic clearance in arthritic joints, prolonging drug retention by 5-fold compared to conventional nanoparticles.^140^ The size of lymphatic vessels and lymphatic nodes shows significant changes during the development of RA, and these changes can be easily detected, suggesting the feasibility of targeting lymphatic vessels for treatment.^68^ One of the more mature aspects of the pathogenesis of OA is the influence of effusion in the disease process. Compared to the findings in RA, OA still has many undefined factors in terms of pathogenesis and therapeutic mechanisms. There are a variety of treatment options available for patients with mild OA, but the mechanisms behind some of the physical therapy options are unclear. The treatment options for patients with severe OA are relatively homogeneous, with total joint replacement being the only effective treatment option. There are many potentially effective therapeutic agents for the treatment of OA, and the clinical outcomes need to be further validated.^8^

TNF therapy: a therapeutic option for RA. Some of the TNF drugs in clinical use restore lymphatic drainage by increasing lymphatic constriction, a process in which there is macrophage apoptosis. This approach contrasts with novel MPO-responsive systems that achieve 3-fold greater reduction in inflammatory cytokines (TNF-α/IL-1β) through lymphatic transport inhibition rather than drainage enhancement.^140^ Targeted local administration is superior to systemically administered therapy.

B-cell depletion therapy: In the development of RA, although its characteristic B-in cells can play a role in slowing down inflammation in the early stage of RA, a large number of proliferating Bin cells will block lymphatic nodes and affect the function of lymphatic drainage with the development of RA. Drugs such as anti-CD20 mAb and rituximab can restore lymphatic drainage by removing the obstruction formed by B-in cells, and the experiments were conducted mainly in animals (mice), and the experiments still showed good results when the drugs were administered locally. Inspired by the fact that the obstruction of lymphatic nodes by B-in cells in RA affects the lymphatic drainage function, the obstruction of lymphatic drainage function in the pathogenesis of OA may also be related to the obstruction caused by B-cells. This therapy is still in the stage of theoretical feasibility in the treatment of OA without any experiments to support it, but it still has some research potential based on conjecture.

VEGF-C/VEGFR3 therapy: Lymphangiectasia in the early stages of RA is dependent on the VEGF-C/VEGFR3 signaling pathway, and lymphangiectasia reduces inflammation in the joints and slows down the progression of RA. VEGF-C adeno-associated viral topical therapy promotes lymphangiectasia to achieve therapeutic effects on RA, and this treatment regimen has already been shown to be effective in RA-modeling mice. Reliable results have been obtained in RA modeling mice. In contrast, in the pathogenesis of OA, although lymphangiogenesis is associated with the VEGF-C/VEGFR3 signaling pathway, the number of mature lymphatic vessels in the synovium surrounding OA appears to decrease at all stages of the disease. The therapy remains controversial and problematic, and the safety of the treatment cannot be guaranteed at this time.

Inhibitors of iNOS: Although lymphatic hyperplasia can slow down the inflammatory process in the early stage of RA, inflammatory factors and iNOS-induced production of NO can damage the surrounding lymphatic vessels to exacerbate the inflammation. Based on this, animal experiments used the iNOS inhibitor L-N(6)-(1-iminoethyl)lysine 5-tetrazole-amide to cause the lymphatic vessels constrict to increase lymphatic drainage and alleviate the inflammation. But at present, some key time points for the use of iNOS inhibitors in the course of the disease treatment are not clear. And in the therapeutic neighborhood of OA, there are many potentially good effects of this approach, but no clinical trial evidence has been seen.

Bortezomib therapy: a therapeutic option for OA. Whether diminished lymphatic drainage accelerates the progression of OA is in speculative stage. As a proteasome inhibitor, the bortezomib drug is still in the stage of animal testing, and its effect of reducing inflammation and improving lymphatic drainage through the NF-κB signaling pathway is currently showing a bright future, which needs to be supported by more experimental and theoretical evidence in the future.^8^

### Targeting the lymphatic vessels as a potential treatment strategy in skeletal muscles

The VEGF-C/VEGFR3 pathway is an important target for targeting lymphatic vessels in skeletal muscle. Lymphatic vessel growth in normal tissues and proliferation in disease states are regulated by the VEGFR3 pathway mediated by VEGF-C. Activation of the VEGF-C/VEGFR3 signaling pathway promotes lymphatic vessel proliferation. In the treatment of skeletal muscle ischemia, injection of VEGF-C into the ischemic muscles of experimental mice can effectively enhance lymphatic drainage and venous return, eliminate muscle tissue inflammation, and improve blood supply conditions. Some researchers envision a combination of vascular endothelial cells and lymphatic vessel targeting agents for the treatment of skeletal muscle ischemia. Animal experiments with human vascular endothelial growth factor (VEGF) family members, vascular endothelial growth factor C/D (VEGF-C/-D), and VEGFR3-specific mutants (VEGF-C156S) and collagen- and calcium-binding transduction of the epidermal growth factor structural domain 1 (CCBE1) promoted lymphatic vascularization, which provides a possible approach for targeting lymphatic vessels for the treatment of skeletal muscle diseases. It is a possible approach that can be used as a reference for studying the site of action of targeted drugs for drug development in the future.^8,10^

### Targeting the lymphatic vessels as a potential treatment strategy in bone

Therapeutic approaches targeting lymphatic vessels are currently not widely used in skeletal diseases, but some of the potential targeting sites identified in the study could serve as a new direction for future research into therapeutic approaches for skeletal diseases.

GSD is a type of CLA in which patients typically develop lymphatic reflux and bones gradually dissolve with the presence of ectopic lymphatic vessels in the bone, and as the disease progresses, celiac disease and other severe bone diseases may develop. The use of trametinib in GSD mice effectively prevents overactive KRAS signaling in LECs from damaging lymphatic valves and has a therapeutic effect of stopping lymphatic reflux. Mouse experiments and case studies have demonstrated that trametinib is an effective GSD therapeutic targeting lymphatic vessel drug, but the specific mechanism of action has not been elucidated.^141^

Acquired HO may occur after orthopedic surgery or be caused by severe trauma, burns, surgery, or central nervous system injury. When mice with acquired HO are given postoperative topical treatment with fibroblast growth factor 9 (FGF9), fibroblast growth factor receptor 3 (FGFR3) signaling, which regulates LECs proliferation, is activated, promoting lymphatic vessel formation and inhibiting HO formation. There is currently no effective treatment for acquired HO, but mouse experiments suggest that FGFR3 is an important target for future treatment of acquired HO by targeting lymphatic vessels.^142^

## Current Challenges and Future Directions

Our understanding of lymphatic vessels in the musculoskeletal system remains incomplete, particularly regarding their structural and functional roles in both health and disease. While the VEGF-C/VEGFR3 signaling pathway has been extensively studied in musculoskeletal disorders,^8–11,139^ it remains unclear whether other molecular pathways contribute similarly to lymphatic regulation. Furthermore, existing research has primarily focused on morphological changes and drainage functions of lymphatic vessels in bones, joints, and skeletal muscles,,^65,143–147^, leaving critical gaps in our knowledge of exercise-induced adaptations and aging-related functional decline. Notably, comparative studies between different muscle groups (e.g., fast vs. slow muscles) are lacking, making it difficult to determine whether lymphatic structure and function vary across musculoskeletal tissues.^145^

The clinical implications of these knowledge gaps are particularly evident in conditions like CLA (congenital lymphatic abnormalities), where therapeutic development has been hampered by an incomplete understanding of pathogenesis.^9^ While research has focused on molecular mechanisms, treatment options remain limited, highlighting the urgent need to translate theoretical knowledge of lymphatic-targeted therapies into experimental validation.

The relationship between lymphatic circulation and immune function in musculoskeletal diseases warrants deeper investigation. While historically believed to be absent from bone tissue,^2^ lymphatic vessels are now recognized as important players in bone homeostasis and repair. Recent advances demonstrate their crucial role in vascularization and bone regeneration following injury,^14^ though this regenerative capacity appears to diminish with age. However, existing detection techniques (e.g., immunofluorescent labeling, single-cell sequencing, and spatial transcriptomics etc.) may still have limitations that affect the accurate knowledge of lymphatic vessels in tissue,s and future technological developments may further optimize the research methods, highlighting the need for improved imaging and analytical techniques.

Emerging evidence suggests complex interactions between bone and lymphatic systems,^148^ with potential implications for both musculoskeletal and neurological disorders. For example, cranial bone manipulation (CBM) has been shown to stimulate meningeal lymphangiogenesis and enhance drainage capacity, potentially reducing amyloid deposition in Alzheimer’s disease. This finding reveals a previously underappreciated mechanism whereby mechanical stimulation of bone can modulate lymphatic function through cytokine release (e.g., VEGF-C) and subsequent endothelial cell activation. Importantly, these effects appear persistent, suggesting long-term structural adaptations in both bone and lymphatic networks. Such discoveries open new avenues for exploring similar bone-lymphatic interactions in musculoskeletal disorders.

The gradual increase in musculoskeletal lymphatic research reflects an evolving understanding of their functional diversity - transitioning from being regarded primarily as passive drainage channels to being recognized as active contributors to tissue homeostasis, repair, and disease modulation.^46,149,150^ This conceptual shift, supported by key discoveries (e.g., the identification of intraosseous lymphatic vessels) and methodological advances (e.g., near-infrared fluorescence imaging with ICG labeling),^85,151^ has enabled preliminary visualization and functional assessment of musculoskeletal lymphatic networks. While these developments have identified potential therapeutic targets for inflammatory joint disorders and trauma repair, substantial work remains to fully characterize these systems. Current methodological progress suggests this field is poised for measured growth in the coming years.

**Fig. 7:**
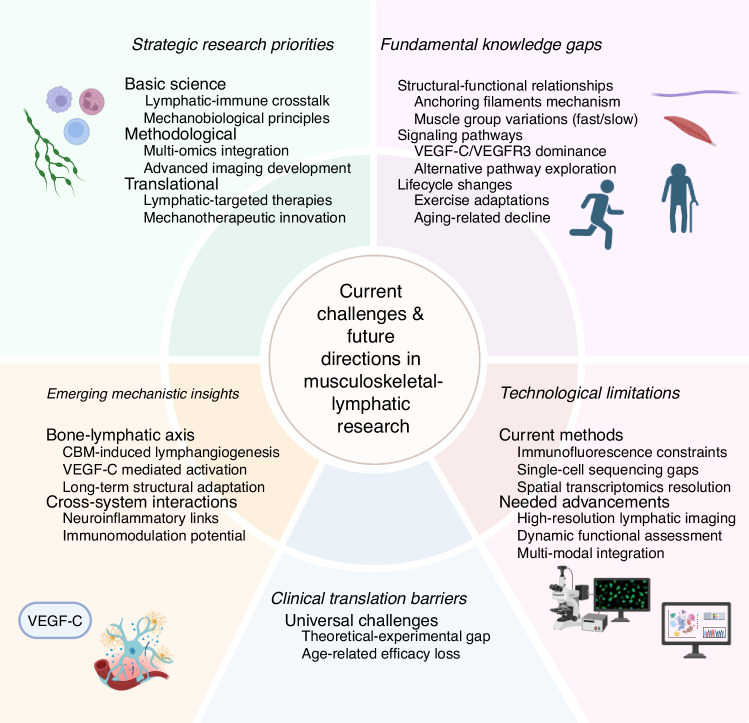
Current Challenges and Future Directions in Musculoskeletal-Lymphatic Research. This conceptual framework summarizes key challenges and emerging opportunities in musculoskeletal-lymphatic research. VEGF-C/VEGFR3 vascular endothelial growth factor C/vascular endothelial growth factor receptor 3, VEGF-C vascular endothelial growth factor C

Future research should focus on elucidating lymphatic-immune crosstalk in inflammatory joint diseases and bone disorders while developing more sophisticated approaches to study musculoskeletal-lymphatic interaction networks. While current evidence has not yet established a direct correlation between bone marrow lymphatic vessels and bone metabolism, substantial research has demonstrated the critical role of bone marrow microenvironment and its cellular components in regulating bone homeostasis and osteoporosis pathogenesis, such as cellular senescence and the inflammatory microenvironment.^152–154^ Considering the specialized functions of lymphatic vessels in immune surveillance and cell trafficking - distinct from those of blood vessels^155,156^ - it is plausible that future investigations may uncover novel relationships between bone marrow lymphatic networks and osteoporosis development. This potential connection warrants further exploration, particularly in light of emerging evidence suggesting lymphatic-immune interactions within the skeletal system.^8,14,85^ In summary, first, the study of the musculoskeletal system can draw on the mechanisms of the lymphatic vascular system in neurodegenerative diseases to explore the potential function of the musculoskeletal system in neuroinflammation and immunomodulation. Second, studies of the lymphatic vascular system can further explore its role in diseases of the musculoskeletal system, particularly the mechanisms of lymphatic drainage and immune cell migration in inflammatory joint diseases and bone disease. In addition, the combination of multi-omics techniques and advanced imaging methods can reveal the interaction network between the musculoskeletal system and lymphatic vascular system and provide a theoretical basis for the development of combined therapeutic strategies targeting disorders of the musculoskeletal system and the lymphatic vascular system. These research directions not only help to deepen the understanding of the physiological and pathological mechanisms of the two major systems, but also provide new perspectives for the comprehensive treatment of cross-system diseases (Fig. 7).

## Conclusion

The lymphatic system has been studied for a long time, and disorders of the lymphatic system have been closely associated with a wide range of diseases, directly or indirectly affecting the homeostasis of the organism, but at the same time providing a possible direction for the cure of diseases. The structure of the lymphatic system at all levels, as well as the functions associated with the structure, are now well defined and clearly understood. However, the initial formation of lymphatic vessels and lymphatic fluid has not yet been clarified, and the role played by anchor wires in the formation of lymphatic fluid is in the conjectural stage. Based on the knowledge of the structure and function of the lymphatic system, a large number of experiments have been conducted to study the distribution of lymphatic vessels in skeletal muscles, joints, and bones. The results of the research on lymphatic vessels in skeletal muscle and joints are more mature and are of great significance in guiding the treatment of related diseases. The discovery of lymphatic vessels in bones breaks through traditional concepts, proves the wide distribution of the lymphatic system in the musculoskeletal system, and provides new ideas for the study of bone diseases (e.g., fracture healing disorders, etc.). In the future, the specific role of the skeletal lymphatic system in bone remodeling and inflammatory response can be further explored using lymphatic tracer technology. Diseases related to the musculoskeletal system, such as RA, muscle ischemia, and muscular dystrophy, have been investigated in terms of their specific pathogenesis and therapeutic methods from the direction of lymphatic vessels. The research results on the pathogenesis and therapeutic methods of OA and CLA in the direction of lymphatic vessels are relatively few, but with a certain theoretical basis, future research has a bright prospect. Lymphatic vascular targeted therapy has shown potential clinical application, but further experiments are needed to verify its efficacy and safety. Future studies should focus on the mechanism of action, the scope of applicable diseases, and the long-term therapeutic effects of lymphatic vessel-targeted drugs, in order to promote the translation of this strategy from the laboratory to the clinic.
